# *Plasmodium falciparum* (Haemosporodia: Plasmodiidae) and O’nyong-nyong Virus Development in a Transgenic *Anopheles gambiae* (Diptera: Culicidae) Strain

**DOI:** 10.1093/jme/tjz032

**Published:** 2019-03-29

**Authors:** John D Mumford, Carole A Long, Scott C Weaver, Katzutoyo Miura, Eryu Wang, Rachel Rotenberry, Ellen M Dotson, Mark Q Benedict

**Affiliations:** 1 Imperial College London, Centre for Environmental Policy, Silwood Park Campus, Ascot, Berkshire, UK; 2 NIH, NIAID, Laboratory of Malaria and Vector Research, Malaria Immunology Section, Twinbrook Pkwy, Rockville, MD; 3 Institute for Human Infections and Immunity and Department of Microbiology & Immunology, University of Texas Medical Branch (UTMB), Galveston, TX; 4 Centers for Disease Control and Prevention, DPDM/Entomology Branch, Atlanta, GA

**Keywords:** competence, risk, transgenic, malaria, arbovirus

## Abstract

Transgenic *Anopheles gambiae* Giles (Diptera: Culicidae) mosquitoes have been developed that confer sexual sterility on males that carry a transgene encoding a protein which cuts ribosomal DNA. A relevant risk concern with transgenic mosquitoes is that their capacity to transmit known pathogens could be greater than the unmodified form. In this study, the ability to develop two human pathogens in these transgenic mosquitoes carrying a homing endonuclease which is expressed in the testes was compared with its nontransgenic siblings. Infections were performed with *Plasmodium falciparum* (Welch) and o’nyong-nyong virus (ONNV) and the results between the transgenic and nontransgenic sibling females were compared. There was no difference observed with ONNV isolate SG650 in intrathoracic infections or the 50% oral infectious dose measured at 14 d postinfection or in mean body titers. Some significant differences were observed for leg titers at the medium and highest doses for those individuals in which virus titer could be detected. No consistent difference was observed between the transgenic and nontransgenic comparator females in their ability to develop *P. falciparum* NF54 strain parasites. This particular transgene caused no significant effect in the ability of mosquitoes to become infected by these two pathogens in this genetic background. These results are discussed in the context of risk to human health if these transgenic individuals were present in the environment.

Considerable progress is being made in reducing malaria transmission using entomological methods, primarily long-lasting insecticide-treated bednets and indoor residual insecticide spraying, along with improved diagnosis and treatment (World Health Organization 2015). A gap still exists between the most optimistic expected outcomes of existing interventions and elimination of malaria (Brown and Rogerson 2016). In addition to the potential obstacles to effectiveness that insecticide and drug resistance present, the shortfall in effort needed for malaria elimination means that unless novel, less expensive and more effective interventions are developed, malaria elimination will remain an aspiration rather than a reality (World Health Organization 2015).

To complement existing interventions, transgenic mosquitoes are being developed in various laboratories that might offer an alternative method at a feasible cost (TDR/WHO 2010). Methods for both population reduction and population replacement (Burt 2014) are envisioned, and considerable progress has been made developing these for *Anopheles gambiae* Giles (Diptera: Culicidae) (Windbichler et al. 2008, Galizi et al. 2014, Hammond et al. 2016) and *Anopheles stephensi* Liston (Diptera: Culicidae) (Isaacs et al. 2011, Gantz et al. 2015), major vectors of human malaria parasites in Africa and Asia, respectively. A potentially powerful and economical method of introducing population suppression and antipathogen effectors into populations is via ‘gene drive’ which spreads transgenes at a rate higher than Mendelian inheritance predicts (Committee on Gene Drive Research in Non-Human Organisms: Recommendations for Responsible Conduct et al. 2016), but its development requires incremental testing of precursor technologies that demonstrate the safety and potential effectiveness of transgenic mosquitoes (WHO/FNIH 2014, Committee on Gene Drive Research in Non-Human Organisms: Recommendations for Responsible Conduct et al. 2016).

As part of this development pathway, a transgenic strain of *A. gambiae* that confers sexual sterility to males has been developed (Windbichler et al. 2008). The effector protein, a fusion of *I-PpoI* DNA nuclease and enhanced green fluorescent protein (*eGFP*), cleaves a 15 bp target site in the 28S ribosomal DNA which consists of hundreds of copies and, in this species, is typically located exclusively on the X chromosome (Collins et al. 1987). The resulting sexual sterility is a trait expressed only in males because the nuclease is controlled by a testes-specific promoter. A sufficient amount of the fusion protein is transported by all sperm produced in transgenic males to cleave the rDNA of the pronucleus so that embryos fail to develop (Windbichler et al. 2008). As a consequence of complete male sexual sterility, the transgene must be maintained in mosquitoes by backcrossing transgenic females to nontransgenic males. This has been done using males of a ‘wild-type’ laboratory strain, in this case one named ‘G3’ (Windbichler et al. 2008, Klein et al. 2012). This process of repeated backcrossing and recombination in both sexes results in progeny in which populations consist of nontransgenic or heterozygous transgenic individuals. Individuals that carry the transgene are genetically distinguishable from those that do not only by the transgene and the immediate vicinity of the chromosome that might not have undergone recombination during the backcrossing process. These nontransgenic individuals provide a useful experimental comparator to test effects specifically due to the transgene in nearly identical genetic backgrounds.

Mating, life history, and sterility characteristics of one particular strain have been extensively studied in the laboratory. The strain, originally named ‘β2Ppo2’ (Windbichler et al. 2008), has since been renamed Ag(DSM)2 by Target Malaria (targetmalaria.org), the research consortium responsible for its development. The strain is sexually sterile (Windbichler et al. 2008, Klein et al. 2012), but males have reduced mating competitiveness (Klein et al. 2012, Facchinelli et al. 2015) and retarded larval development (Klein et al. 2012) compared to nontransgenic siblings.

Assessing risk of these mosquitoes in the environment considers changes in the transgenic mosquito that could lead to harm in comparison to an unmodified form. Among changes that might be considered are changes in behavior, life history, and vectorial capacity. It is the latter that is relevant to this investigation.

We measured characteristics of vector competence for two relevant pathogens, *Plasmodium falciparum*, a causative agent of malaria, and o’nyong-nyong virus, which causes epidemics of a severe febrile, arthralgic syndrome often confused with dengue fever. O’nyong-nyong (ONNV) is one of the few viruses pathogenic to humans that *A. gambiae* is capable of transmitting (Sim et al. 2014). In this report, transgenic and highly related nontransgenic sibling females were compared for their ability to develop these pathogens. The results are presented in the context of risk assessment in public health.

## Materials and Methods

### Mosquito Culture

Transgenic (TR) Ag(DSM)2 females were backcrossed to G3 strain males (MRA-112, BEI Resources) to maintain the transgenic strain. This produced nontransgenic and heterozygous transgenic progeny in each generation with a normal sex ratio (1:1) and a 1:1 proportion of transgenic offspring, which is typical for this dominant Mendelian trait (Windbichler et al. 2008, Klein et al. 2012). We will refer to the nontransgenic progeny as wild type (WT). Comparisons were made between TR and WT sibling females resulting from the same backcross.

Mosquitoes were cultured at the insectaries of the Centers for Disease Control and Prevention (CDC) Entomology Branch in Atlanta, GA, USA. The recorded air temperature and relative humidity (RH) in the insectaries were 27.4°C and 80.4% RH (SD 0.78 and 7.0, respectively), and larvae were cultured according to the standard operating procedure described in the supplemental material of Valerio et al. (Valerio et al. 2016a) using a larval diet designed for use in mosquitoes (Damiens et al. 2012). For routine stock-keeping purposes, adult females were bloodfed on an anesthetized rabbit and all adults were provided with 10% sucrose in water containing 0.1% methylparaben (Benedict et al. 2009). Use of this antimicrobial is the typical stock-keeping procedure in the CDC insectary. Transgenic and nontransgenic individuals were identified in the larval stage based on the 3X-P3 DsRed fluorescent transgene marker and males and females were separated in the pupa stage based on genitalia. Adults that were shipped for experimental infections were provided 10% sucrose in water that did not contain methylparaben to ensure that the antimicrobial activity did not affect pathogen development. Prior to shipping, females were allowed to mate with the sexually sterile Ag(DSM)2 transgenic males as a containment precaution. They were shipped in 9 cm diameter, 10 cm high polyethylene cups covered with screen tops and provided with 10% sucrose in containers approved for shipping transgenic mosquitoes (UN 3245). Only females were shipped and transit time was overnight. Female mosquitoes were 4–7-d old upon arrival at NIH or UTMB, and infections were performed the same or the following day.

### ONNV Infections

Experimental ONNV infections were performed at the University of Texas Medical Branch in Galveston, TX, USA under arthropod containment level 2 conditions. ONNV strain SG650 was isolated from human serum in Uganda in 1996, passed twice in Vero cells and stored at −80°C for use in all experiments. A stock of titer 4.4 × 10^6^ Vero cell plaque-forming units was used for mosquito infections.

#### Intrathoracic infections

Cohorts of 30–50 adult TR and WT females were injected intrathoracically, using glass capillary pipettes heated and pulled to fine tips, with 1–2 µl of 0, 1, 2.5, and 3.5 Log_10_ plaque-forming units (PFU)/ml stocks of ONNV infection dose (titers confirmed by back-titration on Vero cells of the stocks after inoculations). Mosquitoes were held for an extrinsic incubation period of 7 d at 27°C and 80% RH before being assayed. Twenty mosquitoes were harvested on day 7 postinfection (PI) for each infection dose. Each infected mosquito was placed in an Eppendorf (Hamburg, Germany) tube with 300 µl of Dulbecco’s modified Eagle’s essential medium (DMEM) with 10% fetal bovine serum (FBS), and amphotericin B (50 μg/ml), triturated for 4 min, and centrifuged. The supernatant from each mosquito was screened for virus content by the induction of cytopathic effects (CPE) on Vero cells.

#### Oral infectious dose, infection, dissemination, and transmission

Four doses of ONNV from 10^2^ to 10^5^ PFU/ml were made by serial 10-fold dilutions. Viruses were mixed with an equal volume of defibrinated rabbit blood and a final concentration of 2 mM ATP. TR and WT mosquitoes were fed in an isolation glove box located in a BL2/ACL2 insectary. Infectious blood was heated to 37°C in a Hemotek feeding apparatus (Discovery Workshops, Accrington, Lancashire, UK) for 1 h. Then, fully engorged mosquitoes were separated from unfed females and were transferred into fresh cartons with 10% sucrose water on cotton for extrinsic incubation at 28°C, a typical mean tropical temperature.

After extrinsic incubation and cold-anesthesia, wings and legs of each mosquito were removed and the proboscis was inserted into a 10 µl capillary tube containing 8 µl of DMEM with10% FBS for 45 min. The mixture of DMEM-FBS and saliva was expelled into 100 µl of DMEM medium and stored at −80°C until further processing.

To compare oral infectious dose 50% values, 30 mosquitoes were harvested on days 7 and 14 postinfection (PI) after each infection dose. Saliva, legs, and remaining mosquito bodies were collected separately. Body infection rates were calculated for oral infectious doses 50% value.

#### Viral assays of infected bodies and legs

From each dose batch, up to 30 potentially infected mosquito bodies and legs were placed in Eppendorf (Hamburg, Germany) tubes with 300 µl of DMEM containing 10% FBS, and amphotericin B (50 μg/ml), triturated for 4 min, and centrifuged. Supernatants from each body were screened for virus content by the induction of CPE on Vero cells. If body samples were positive, indicating initial oral infection, viral titers of bodies and legs (a proxy for dissemination into the hemocoel) were determined by plaque assay in Vero cells. If legs were positive, indicating a disseminated infection, saliva was assayed for CPE on Vero cells to detect ONNV.

### *P. falciparum* infections

*Plasmodium falciparum* infections were performed at the National Institutes of Health, Malaria Immunology Section in Bethesda, MD, USA. All infections were performed within BL2/Arthropod Containment Level 2 conditions. The gametocyte culture of the *P. falciparum* NF54 line and standard membrane-feeding assay (SMFA) were performed as described previously (Miura et al. 2013) with three modifications. In this study, WT and TR mosquitoes were utilized in addition to *A. stephensi* mosquitoes (a known susceptible species, to check on gametocyte uptake success). Second, all mosquitoes were fed gametocyte mixtures in normal human sera (without any transmission-blocking antibodies). Lastly, midguts from all mosquitoes with any eggs in their ovaries at the time of dissection (8 d after feed) were analyzed (instead of *n* = 20 per group). The stage V gametocytemia in feeds 1, 2, and 3 were 1.88, 0.69, and 1.14%, respectively. Two different batches of pooled human sera (pools of 20 or 39 individual sera) were used in this study (one pool for feed 1 and 2, and the other pool for feed 3). Different batches of red blood cells were used for different feeding experiments. The human serum and RBC used in this study were purchased from Interstate Blood Bank, Inc. (Memphis, TN).

### Statistics

In all statistical tests, the alpha was 0.05. Fisher’s exact probability test was used to compare the ONNV intrathoracic and oral infections. Tukey’s multiple comparison test was used for body part infection comparisons. Fisher’s exact probability test was used to analyze the proportion of mosquitoes infected (prevalence). The best estimate of oocyst ratio between WT and TR, the 95% confidence intervals (95% CI), and *P*-values (whether there was a significant difference in oocyst density between WT and TR) from single or multiple feeds were calculated using a zero-inflated negative binomial random effect model, which was generated from a large SMFA data set along with *A. stephensi* mosquitoes (Miura et al. 2013).

## Results

### ONNV Infections

#### Intrathoracic infections

**Fig. 1.**
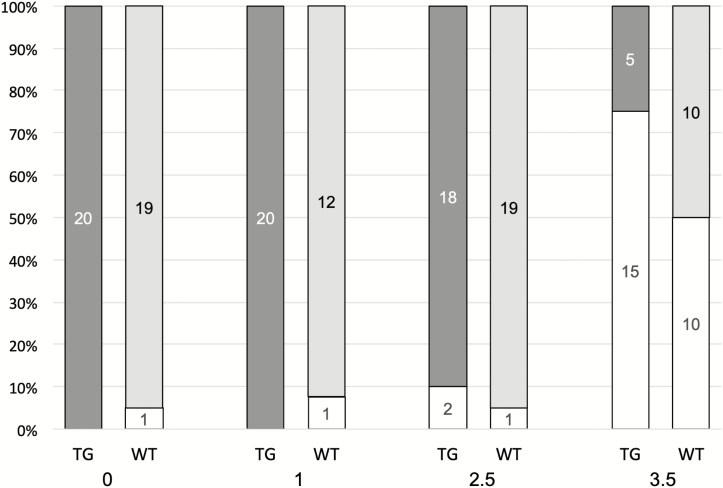
Number infected females after intrathoracic injection of ONNV. The back titer doses (Log_10_ PFU/ml) are shown beneath each set of infections. Shaded bars indicate the females that were uninfected.

To examine potential differences in ONNV replication within the two mosquito strains, cohorts of both mosquito strains were inoculated intrathoracically. The results are shown in Fig. 1. No significant difference in the number of females that became infected was observed at any dose between the transgenic and wild-type populations. Fisher’s exact test probabilities for the four doses tested (0, 1, 2.5, 3.5 back titer dose (Log_10_ PFU/ml) were 0.50, 0.39, 0.38, and 0.07, respectively).

#### ONNV oral infectious doses 50% value, rates of infection, dissemination, and saliva

Because another goal of the study was to determine if the two mosquito strains differed in oral susceptibility, doses that generated intermediate infection rates that would allow us to detect vector competence differences were intentionally used. Following the ingestion of infectious blood meals, there were no significant differences (*P* > 0.05, Fisher’s exact test) between TR and WT comparator mosquitoes in the numbers of females that became infected (ONNV-positive bodies), the number that had detectable virus in the legs (dissemination from the midgut into the hemocoel) or saliva at 7 d PI (Table 1): all saliva tested negative. Although there were no positive controls for salivation, prior studies have shown rates >90% with these methods (S.C.W., unpublished data). For the 7 d PI bodies, the oral ID_50_ for the TR and WT were Log_10_=4.10 and Log_10_=3.74, respectively.

**Table 1.** Results of oral infection with ONNV in *A. gambiae* mosquitoes at 7 d PI expressed as the number infected/total number that fed and survived incubation (% positive)

Blood meal titer (Log_10_ PFU/ml)	Body			Legs			Saliva	
	TR	WT	*P* ^*^a^*^	TR	WT	*P*	TR	WT
1.9	0/30 (0)	0/30 (0)	1.00	ND^*b*^	ND		ND	ND
3.0	1/30 (3.3)	3/30 (10.0)	0.25	0/1 (0)	0/3 (0)	1.00	ND	ND
4.6	8/29 (27.6)	13/30 (43.3)	0.09	0/8 (0)	2/13 (15.4)	0.37	ND	0/2 (0)
5.8	24/30 (80.0)	28/30 (93.3)	0.10	1/24 (3.6)	0/28 (0)	0.46	0/1 (0)	ND

^*a*^Fisher’s Exact Probability

^*b*^Not done. The analysis is progressive; only females that were positive for body infection were analyzed for leg infection and of those, only those that were positive for leg infection were analyzed for virus in saliva.

The same measures were made at day 14 PI (Table 2). For the 14 d PI bodies, the oral ID_50_ for the TR and WT were Log_10_ = 3.12 and Log_10_ = 3.46, respectively. Two saliva samples were positive by CPE assay in the TR mosquito group at the highest dose (5.6 Log_10_). All saliva samples were negative in WT mosquito groups. Overall, our infection and dissemination results showing limited vector competence were similar to those reported previously (Brault et al. 2004) with this ONNV strain in *A. gambiae*, and there was no significant difference in infection rate between TR and WT groups at any dose.

**Table 2.** Results of oral infection with ONNV in *A. gambiae* mosquitoes at 14 d PI expressed as the number infected / total number that fed and survived incubation (% positive)

Blood meal titer (Log_10_ PFU/ml)	Body			Legs			Saliva		
	TR	WT	*P* ^*^a^*^	TR	WT	*P*	TR	WT	*P*
2.4	0/30 (0)	0/30 (0)	1.00	ND^*b*^	ND		ND	ND	
3.3	4/30 (13.3)	2/30 (6.7)	0.24	2/4 (50.0)	½ (50.0)	0.60	0/2 (0)	ND	
4.1	22/30 (73.3)	18/30 (60.0)	0.12	11/22 (50.0)	8/18 (44.4)	0.23	0/11 (0)	0/2 (0)	1.00
5.6	24/30 (80.0)	26/30 (86.7)	0.21	20/24 (83.3)	17/26 (65.4)	0.09	2/20 (10.0)	ND	

^*a*^Fisher’s Exact Probability

^*b*^Not done. The analysis is progressive; only females that were positive for body infection were analyzed for leg infection and of those, only those that were positive for leg infection were analyzed for virus in saliva.

#### Viral concentration of infected bodies and legs

No significant differences were found among mean body viral titers; however, there were significant differences in leg titers between the 4.1 Log_10_ dose of the TR mosquito strain and the 5.6 Log_10_ dose treatment in the WT strains strain and between the 5.6 Log_10_ highest dose treatment of the TR and WT strains (Table 3). Note that the number of individuals that were positive for CPE is greater than the number of individuals from which a plaque titer was obtained because of the higher sensitivity of the CPE screening assay. The high-dose WT leg sample had a particularly low proportion of individuals in which the virus titer could be detected, which may have affected the statistical interpretation. Two saliva samples were positive by CPE assay in the TR mosquito group at the highest dose (5.6 Log_10_) but the titer was below the detection limit for the plaque assay. All saliva samples were negative by CPE in WT mosquito groups.

**Table 3.** Mean titers per sample of *A. gambiae* mosquitoes orally infected with ONNV at days 14 PI ± standard deviation (number analyzed)

Blood meal titer (Log_10_ pfu/ml)	Body^*^a^*^		Legs	
	TR	WT	TR	WT
2.4	Not detectable in either body part			
3.3	4.2 (4)	3.1 (1)	4.4 (1)	2.5 (1)
4.1	4.3 ± 0.8 (17)	3.9 ± 1.0 (18)	4.4 ± 0.5^*^b^*^ (9)	3.8 ± 0.6 (5)
5.6	4.7 ± 0.8 (24)	4.3 ± 0.7 (26)	4.3 ± 0.5^*^c^*^ (19)	3.2 ± 0.5^*^b,c^*^ (7)

^*a*^No significant differences were detected for mean body viral titers (Tukey’s Multiple Comparison Test, *P* > 0.1 for all comparisons).

^*b*^Mean leg titers were significantly different between 4.1 Log_10_ dose for TR vs 5.6 Log_10_ dose for WT (*P* < 0.001)

^*c*^Mean leg titers were significantly different between 5.6 Log_10_ dose TR vs 5.6 Log_10_ dose for WT (*P* < 0.001).

### *P. falciparum* Infections

Three *P. falciparum* infections were performed. As an infection control, simultaneous infections of *A. stephensi*, which were reared at NIH, were performed, and the mean oocysts per mosquito in feedings #1, #2, and #3, were 56, 26, and 56, respectively, showing satisfactory uptake in the feeds. The results of two groups of *A. gambiae*, both of which were cultured at CDC and shipped to NIH for SMFA testing at the same time, are presented in Table 4.

**Table 4.** *P. falciparum* infection intensity and prevalence

Blood feeding	Infection intensity				Infection prevalence			
	Mean oocysts/ female (*n*, SD)		One-tail *t*-test *P*		Number females infected/total that fed (%)		*P*	
	WT	TR			WT	TR		
1	7.94 (47, 15.65)	9.16 (57, 17.96)	0.356	(WT=TR)	27/47 (51.1)	34/57 (59.6)	0.843	(WT=TR)
2	2.04 (49, 3.99)	0.63 (63, 2.88)	0.020	(WT>TR)	18/49 (36.7)	8/63 (12.7)	0.003	(WT>TR)
3	7.72 (76, 9.57)	18.5 (114, 21.94)	3.736E-06	(WT<TR)	62/76 (81.6)	106/114 (93.0)	0.021	(WT<TR)

With regard to prevalence of infection, the three SMFAs each gave different relative results. In blood-feeding number 1, the prevalence was not significantly different between the two groups. In one of the other two (number 2), the WT group had higher prevalence and in number 3, the WT prevalence was lower. Mirroring these results, there was no significant difference in oocyst intensity in blood-feeding number 1 between TR and WT (*P* = 0.665), while there were significant differences in feeding 2 (*P* = 0.002, WT > TR) and feeding 3 (*P* = 0.007, WT < TR). When all three feeding results were analyzed together, there was no significant difference in oocyst intensity: the best estimate of oocyst intensity ratio (oocyst intensity in TR over that in WT) = 0.95 (95% CI, 0.63–1.4) and *P* = 0.801.

## Discussion

With regard to two important pathogens transmitted by *A. gambiae*, we observed few differences between WT and TR mosquitoes. ONNV virus was rarely detected in the saliva even after ingestion of large oral doses and 14 d of extrinsic incubation. The rate of saliva infection, the critical measure of vector competence, did not differ significantly between WT and TR mosquitoes. Apart from titers in infected mosquito legs following medium and high-dose blood meals, there was no evidence of a significant difference in the susceptibility, permissiveness for viral dissemination or for replication of ONNV between the WT versus TR populations. The ONNV titer in legs after oral infection with high dose was very low in the WT pooled leg sample, with a significantly lower mean than for the TR group at both of the higher doses. No significant difference was seen between WT and TR in the body samples at any dose. Although the logistic challenges of rearing mosquitoes in one location and performing infections in another precluded replicates of experiments, our data indicate no meaningful difference between the two mosquito strains in vector competence.

Similarly, the absence of consistent differences observed in the prevalence and intensity of *P. falciparum* infection in each assay. When data from all three assays are combined, the oocyst ratio between TR and WT was 0.95 (95% CI, 0.63–1.4). If the assays were performed with higher mean oocysts and/or more mosquitoes were dissected per assay, or if more than three repeat feeds were performed, the range of 95% CI could become narrower. However, the insignificant result (*P* = 0.8) suggests that even if Ag(DSM)2 females were released into the environment, they would pose no additional risk to a release of the same number of wild-type females with regard to this characteristic.

These results and similar laboratory assays of components of vector competence cannot fully capture the range and variation in response that might be encountered naturally. Whereas laboratory experiments such as these are conducted under controlled conditions to increase the experimental sensitivity to differences, a wider range of temperatures and genetic backgrounds would be experienced by transgenic individuals in the environment, both of which might affect vector competence. The results do provide an indicator of whether concern is warranted.

It is not apparent that there is any mechanism by which this particular transgene could affect the ability of mosquitoes that carry it to develop viruses or parasites. We are aware of only one report (Pompon and Levashina 2015) of a common pathway between immunity and sexual sterility. In that report, an allele of an immune factor TEP1 that is known to affect *A. gambiae* susceptibility to malaria parasites is also recruited to eliminate sperm in males that have DNA breakage due to irradiation. The Ag(DSM)2 strain has DNA breakage due to rDNA cleavage and it has also been observed to have morphologically defective sperm in the testes and spermatheca (C. Oliva, data not shown and personal communication) which might recruit this protein. In the experiments performed here, both TR and WT mosquitoes were mated to TR males and would have received a similar amount of TEP1 in the sperm. However, there is no information to indicate that this mode of transfer or amount would be likely to affect susceptibility of females.

Ag(DSM)2 is a sterile male strain that is intended only for small scale field study in Africa as a step in progressive biosafety and entomological testing, subject to regulatory approval. The intended releases that have now been approved by the National Biosafety Authority in Burkina Faso (approval number 2018–453) would consist predominantly of males, and the number of females that has been approved would be less than 25 individuals. Natural village level populations of *Anopheles* mosquitoes in Burkina Faso are estimated at 100,000–500,000 males (with similar numbers of females) in a village of approximately 1 km^2^ (Epopa et al. 2017). The release protocol involves intensive manual sorting to select males, however, even if a small number of transgenic females occur in the releases, due to low fitness characteristics in mating competitiveness and survival, the persistence of the trait in females in generations after release is expected to be very short (Valerio et al. 2016b). Differences in infection rates due to any differences in vectorial capacity within the limited ranges shown in these tests would be undetectable in a natural population with the numbers of transgenic females potentially present.

## Declarations

Ethics approval and consent to participate

No human subjects participated in this research. Blood-feeding on anesthetized rabbits was approved under a CDC permit 2863-DOTRABC-A2.

## Consent for Publication

All authors have read and approved the final version of this manuscript.

## Availability of Data and Material

The datasets used and/or analyzed during the current study are available from the corresponding author on reasonable request.

## Competing Interests

J.D.M. is responsible for risk analysis within the Target Malaria project. M.Q.B. is a consultant for the Target Malaria project which funded the development of these strains. E.M.D. is a collaborator on the Target Malaria project.

## Authors’ Contributions

M.Q.B. and J.D.M. conceived the overall studies and J.D.M. conducted the statistical analysis. S.C.W. and C.A.L. designed and supervised the ONNV and *P. falciparum* laboratory studies. K.M. conducted the technical work with parasites and E.W. with the virus infections). R.R. cultured mosquitoes and prepared shipments. M.Q.B. oversaw drafting of the manuscript and author coordination. E.M.D. provided oversight of staff and assisted drafting and editing the manuscript.

## Funding

Target Malaria receives core funding from the Bill & Melinda Gates Foundation and from the Open Philanthropy Project Fund, an advised fund of Silicon Valley Community Foundation. The works performed at National Institute of Allergy and Infectious Diseases (NIAID) were supported by Division of Intramural Research, National Institute of Allergy and Infectious Diseases, NIH.
